# Hypoxia-responsive circular RNA *circAAGAB* reduces breast cancer malignancy by activating p38 MAPK and sponging *miR-378 h*

**DOI:** 10.1186/s12935-023-02891-0

**Published:** 2023-03-10

**Authors:** Kuan-Yi Lee, Chia-Ming Liu, Li-Han Chen, Chien-Yueh Lee, Tzu-Pin Lu, Li-Ling Chuang, Liang-Chuan Lai

**Affiliations:** 1 Graduate Institute of Physiology, College of Medicine, National Taiwan University, Taipei, Taiwan; 2 Institute of Fisheries Science, College of Life Science, National Taiwan University, Taipei, Taiwan; 3 Master Program for Biomedical Engineering, College of Biomedical Engineering, China Medical University, Taichung, Taiwan; 4 Institute of Epidemiology and Preventive Medicine, Department of Public Health, National Taiwan University, Taipei, Taiwan; 5 School of Physical Therapy and Graduate Institute of Rehabilitation Science, College of Medicine, Chang Gung University, Taoyuan, Taiwan; 6 Department of Physical Medicine and Rehabilitation, Chang Gung Memorial Hospital at Linkou, Taoyuan, Taiwan; 7 Bioinformatics and Biostatistics Core, Center of Genomic and Precision Medicine, National Taiwan University, Taipei, Taiwan; 8 Department of Life Science, College of Life Science, National Taiwan University, Taipei, Taiwan

**Keywords:** *circAAGAB*, Hypoxia, Breast cancer, Tumor suppressor, p38 MAPK, miRNA sponge, Radiosensitivity

## Abstract

**Background:**

Breast cancer is a prevalent disease in women, with high prevalence worldwide. The hypoxic microenvironment of solid tumors develops during the progress of carcinogenesis and leads to greater malignancy and treatment resistance. Recently, accumulating evidence indicates that non-coding RNAs, such as circular RNAs (circRNAs), play a pivotal role in altering cellular functions. However, the underlying mechanisms of circRNAs in breast cancer are still unclear. Therefore, the purpose of this study was to investigate the role of a tumor-suppressive circRNA, *circAAGAB,* in breast cancer by assuming down-regulation of *circAAGAB* under hypoxia and the properties of a tumor suppressor.

**Methods:**

Firstly, *circAAGAB* was identified from expression profiling by next generation sequencing. Next, the stability of *circAAGAB* increased by interacting with the RNA binding protein FUS. Moreover, cellular and nuclear fractionation showed that most *circAAGAB* resided in the cytoplasm and that it up-regulated *KIAA1522*, *NKX3-1*, and *JADE3* by sponging *miR-378 h*. Lastly, the functions of *circAAGAB* were explored by identifying its down-stream genes using Affymetrix microarrays and validated by in vitro assays.

**Results:**

The results showed that *circAAGAB* reduced cell colony formation, cell migration, and signaling through p38 MAPK pathway, as well as increased radiosensitivity.

**Conclusion:**

These findings suggest that the oxygen-responsive *circAAGAB* acts as a tumor suppressor in breast cancer, and may contribute to the development of a more specific therapeutic regimen for breast cancer.

## Introduction

Cancer is a major disease worldwide with high occurrence, poor prognosis, and high mortality [1]. Because of genetic and epigenetic changes, normal cells progressively transform into cancer cells, resulting in uncontrolled cell division and rapid growth [2]. Two main categories of genes are involved in the process of carcinogenesis: oncogenes and tumor suppressor genes. Tumor suppressor genes can inhibit cell proliferation and tumor development in normal cells. However, when tumor suppressor genes are inactivated by loss of function mutations, they facilitate tumorigenesis [3]. During solid tumor progression, the rapid proliferation of cancer cells outpaces the growth of the surrounding blood vessels and results in insufficient blood supply, leading to a hypoxic microenvironment. Thus, cancer cells must alter their molecular mechanisms and metabolism to adapt to hypoxia in order to support continuous growth and proliferation.

In addition, due to these genetic alterations, tumor hypoxia enhances resistance to treatments such as chemotherapy, radiotherapy, and immunotherapy. Hypoxia decreases pH and forms an acidic microenvironment, which leads to drug resistance through a series of mechanisms, such as a lower concentration of the drug caused by the ion trapping phenomenon [4] or activity of a multidrug transporter p-glycoprotein [5]. Also, hypoxia enhances resistance to radiation therapy [6]. Thus, most hypoxic tumor cells grow continuously. Furthermore, immunity is also suppressed in a hypoxic microenvironment by inhibiting the recruitment of T-cells, myeloid-derived suppressor cells, macrophages, and neutrophils [7], or increasing resistance of tumor cells to the cytolytic activity of immune effectors [8, 9], as well as up-regulating immune checkpoint proteins, such as programmed death 1 ligand (PD-L1) [10] and cytotoxic T-lymphocyte-associated protein 4 (CTLA-4) receptor [11]. Altogether, tumor hypoxia makes cancer cells more malignant and resistant to therapy.

A growing body of evidence shows that non-coding RNAs play a pivotal role in regulating signaling pathways and modulating tumor progression [12, 13]. Circular RNAs (circRNAs), a class of non-coding RNAs with a single-stranded circular structure, were considered as functionless byproducts of aberrant RNA splicing at first [14]. In fact, they are functional nucleic acids derived from pre-mRNA and created through back-splicing of the 3’ end of a donor to the 5’ end of an acceptor [15]. This particular form enables circRNAs to lack polyA tails and be more stable than linear RNAs [16]. Recently, a number of reports have indicated that circRNAs have many biological functions [17]. For example, circRNAs act as a sponge for microRNA (miRNA) to inhibit its interaction with its target genes in the cytoplasm [18–20]. CircRNAs also interact with RNA binding proteins (RBPs) to regulate transcription [21–23], splicing, or epigenetic alterations [21, 23–25]. Some research also demonstrated that circRNAs can be translated into functional small peptides through ribosome binding on internal ribosome entry site (IRES) [26, 27]. As a result of these diverse functions, circRNAs are involved in the pathogenesis of various human diseases, such as cardiovascular disease [28], diabetes [29], nervous system disorders [30], and cancer [31]. Especially, circRNAs play the crucial roles of oncogene or tumor suppressor via a variety of mechanisms. For instance, an oncogenic circRNA, *circRNA-MYLK*, induced epithelial–mesenchymal transition (EMT), cell proliferation, and angiogenesis by activating the VEGFA/VEGFR2 signaling pathway in bladder cancer [32]. In contrast, *cir-ITCH* acted as a tumor suppressor in colorectal cancer by regulating the Wnt/β-catenin pathway [33]. However, the detailed mechanism of circRNA in regulating breast cancer cells as they adapt to hypoxia still remains unclear.

In this study, a hypoxia-responsive circRNA, *circAAGAB*, derived from the alpha- and gamma-adaptin-binding protein p34 gene *AAGAB,* was identified in breast cancer MCF-7 cells by RNA sequencing, and the circular structure and expression levels under different oxygen concentrations were validated. *CircAAGAB* resided mainly in the cytoplasm and was found to sponge the miRNA *miR-378 h* and bind to the RNA binding protein FUS. Finally, genes regulated by *circAAGAB* were identified by Affymetrix microarrays, and in vitro functional assays showed inhibition of proliferation and migration ability as well as the increase of radiosensitivity in breast cancer MDA-MB-231 cells overexpressing *circAAGAB*.

## Materials and methods

### Cell culture

Breast cancer cell lines, MCF7 and MDA-MB-231, and the HEK293T cell line were cultured in Dulbecco’s Modified Eagle Medium (GIBCO, Carlsbad, CA, USA). MDA-MB-361 cells were cultured in L-15 medium (GIBCO). ZR-75-30 cells were cultured in Dulbecco's Modified Eagle Medium: Nutrient Mixture F-12 (GIBCO). T47D cells were cultured in RPMI 1640 medium (GIBCO). All breast cancer cell lines and HEK293T were supplemented with 10% fetal bovine serum (FBS) (HyClone, Logan, UT, USA) and 1% penicillin–streptomycin solution (GIBCO). All cell lines were incubated at 37 °C in a humidified incubator with 5% CO_2_ under normoxia. To verify whether *circAAGAB* was oxygen-responsive, cells were cultured in a hypoxic chamber (InVivO2-200, Ruskinn Technology, Bridgend, UK) filled with 0.5% O_2_, 5% CO_2_, and 94.5% N_2_ for 24 h. After incubation under hypoxia, cells were moved to the humidified incubator with normoxic conditions for another 24 h to mimic re-oxygenation.

### Cell line authentication

Cell experiments were performed on cells that were passaged less than 15 times and were routinely tested for mycoplasma using PCR Mycoplasma Detection Kit (ABM Inc., Vancouver, Canada). The cell lines were purchased and authenticated by the Bioresource Collection and Research Center, Food Industry Research and Development Institute (Hsinchu, Taiwan).

### Plasmid construction, RNA interference, and miRNA overexpression

To overexpress circular RNA *circAAGAB*, the *circAAGAB* sequence was inserted into the DNA plasmid pCIRC2T7 to construct pCIRC2T7-*circAAGAB* (BioMed Resource Core (BMRC) of the 1st Core Facility Lab, College of Medicine, National Taiwan University). To check the interaction and binding site between *circAAGAB* and *miR-378 h*, plasmid pMIR-REPORT-*circAAGAB* (BMRC) was constructed by inserting the *circAAGAB* sequence behind the sequence of firefly luciferase. In addition, pMIR-REPORT-*circAAGAB*-mut (BMRC) was constructed by mutating the putative binding site on *circAAGAB*. To knock down the expression of FUS, MDA-MB-231 cells were transfected with 5 μM of small interfering RNA (siRNA) (Dharmacon, Lafayette, CO, USA) in 2 mL medium for 48 h. To overexpress *miR-378 h*, MDA-MB-231 cells were transfected with 5 μM of *miR-378 h* mimics (Dharmacon) in 2 mL medium for 48 h. After transfection, MDA-MB-231 cells were lysed to extract total RNA.

### Genomic DNA extraction, RNA isolation, reverse transcription, and quantitative RT-PCR

Genomic DNA (gDNA) was extracted by QIAamp DNA Kits (Qiagen, Hilden, Germany). Cells were lysed by Nucleozol reagent (Machery-Nagel, Düren, Germany) and total RNA was purified according to the manufacturer’s protocols. Subsequently, total RNA was reverse-transcribed to complementary DNA (cDNA) using a High-Capacity cDNA Reverse Transcription Kit (Applied Biosystems, Carlsbad, CA, USA). For reverse transcription of miRNA, SuperScript IV Reverse Transcriptase (Invitrogen, CA, USA) was used with the primers (Table 1). cDNA acted as the template for quantitative RT-PCR with OmicsGreen qPCR MasterMix (OmicsBio, New Taipei City, Taiwan), and the cycle threshold (Ct) value was detected by StepOnePlus Real-Time PCR System (Thermo Fisher, Waltham, MA, USA). The relative gene expression was evaluated using the 2^−∆∆Ct^ method.

**Table 1:** The primers for quantitative RT-PCR

Gene/miRNA		Primer	Sequence (5ʹ to 3ʹ)	
Reverse transcription				
* miR-3127-5p*		GTTGGCTCTGGTGCAGGGTCCGAGGTATTCGCACCAGAGCCAACCTTCCCA		
* miR-671-5p*		GTTGGCTCTGGTGCAGGGTCCGAGGTATTCGCACCAGAGCCAACCTCCAGC		
* miR-422a*		GTTGGCTCTGGTGCAGGGTCCGAGGTATTCGCACCAGAGCCAACGCCTTCT		
* miR-378i*		GTTGGCTCTGGTGCAGGGTCCGAGGTATTCGCACCAGAGCCAACCCTTCTG		
* miR-378 h*		GTTGGCTCTGGTGCAGGGTCCGAGGTATTCGCACCAGAGCCAACCCATCTG		
* U6* snRNA		CGCTTCACGAATTTGCGTGTCAT		
Quantitative RT-PCR				
* circAAGAB*		Forward	CTGAATGCCAATGTGTGGTC	
		Reverse	CTATCAAGGCCCGATTTTTG	
* AAGAB*		Forward	ACAGCACACAAA AATCGGGC	
		Reverse	ATTGGCATTCAGGGCTTGGA	
* AAGAB* exon 2		Forward	AGTGACTTCCAATGATGCTGT G	
		Reverse	TGTGTGCTGTCAAAGTAAACCAC	
* 18S* rRNA		Forward	TCAACTTTCGATGGTAGTCGCCGT	
		Reverse	TCCTTGGATGTGGTAGCCGTTTCT	
* GAPDH*		Forward	AACGGGAAGCTTGTCATCAATGGA AA	
		Reverse	GCATCAGCAGAGGGGGCAGAG	
* BCAR4*		Forward	GTTCCGATGCTTGTCTTGCTC	
		Reverse	CCAAAGACGAAGATGCCAGG	
* U6* snRNA		Forward	GCTTCGGCAGCACATATACTAAAAT	
		Reverse	CGCTTCACGAATTTGCGTGTCAT	
* miR-3127-5p*		Forward	CGATCAGGGCTTGTGGAA	
		Reverse	GTGCAGGGTCCGAGGT	
* miR-671-5p*		Forward	CGGAGGAAGCCCTGGA	
		Reverse	GTGCAGGGTCCGAGGT	
* miR-422a*		Forward	CGCGACTGGACTTAGGGTC	
		Reverse	GTGCAGGGTCCGAGGT	
* miR-378i*		Forward	CGCGACTGGACTAGGAGT	
		Reverse	GTGCAGGGTCCGAGGT	
* miR-378 h*		Forward	CGCACTGGACTTGGTGT	
		Reverse	GTGCAGGGTCCGAGGT	
* KIAA1522*		Forward	CCAGGACAACGTCTTCTTTCC	
		Reverse	CAGCCACCCTTGTTCAGTTTC	
* NKX3-1*		Forward	CCCACACTCAGGTGATCGAG	
		Reverse	GAGCTGCTTTCGCTTAGTCTT	
* PHC3*		Forward	ACAGCAGTCAAGTATGTCCCA	
		Reverse	CTTGCCGTTAGGGTAGGGG	
* ELAC1*		Forward	GCTGGTCTTCCATTATGTGGTT	
		Reverse	GTGAAAGAGGCGAAATGCTTTT	
* ZNF124*		Forward	AAAGCCTTAGGTTTTTCCCGTT	
		Reverse	ACATGGATAGGGTTCTTCACCA	
* RAB10*		Forward	CTGCTCCTGATCGGGGATTC	
		Reverse	TGATGGTGTGAAATCGCTCCT	
* KCNIP2*		Forward	AATGTCCCAGCGGAATTGTCA	
		Reverse	GAGCCATCATGGTTGGTGTCA	
* PLCXD2*		Forward	GACTCGTTTCTTACACAGCACC	
		Reverse	AGCGTCAAACTTTCCACACTG	
* JADE3*		Forward	GACGTTCTGTTTATCCGACCC	
		Reverse	CCACAACCCATTTCTGCAAGG	
* FUS*		Forward	AGCTGGTGACTGGAAGTGTC	
		Reverse	GGTAGCCGCCTCGATCATAG	
* EGR1*		Forward	CCAGTGGAGTCCTGTGATCG	
		Reverse	TTCATCGCTCCTGGCAAACT	

### Western blotting

MDA-MB-231 cells were lysed in RIPA lysis buffer (Millipore, Billerica, MA, USA) with 0.1% SDS and sonicated. Subsequently, the proteins diluted by sample buffer were separated by sodium dodecyl sulfate‑polyacrylamide gel electrophoresis (SDS-PAGE) and then transferred onto polyvinylidene difluoride (PVDF) membranes (Bio‑Rad, Hercules, California, USA). The membranes containing proteins were blocked in Lightning Blocking Buffer (ArrowTec Life Science, Taiwan) for 5 min. Afterwards, membranes were incubated with primary antibodies against FUS (cat. no. A5921; ABclonal, Woburn, MA, USA), EGR1 (cat. no. 4154; Cell Signaling, Danvers, MA, USA), ATF3 (cat. no. 18665; Cell Signaling), phospho-p38 MAPK-T180/Y182 (cat. no. AP0526; ABclonal), total p38 MAPK (cat. no. A4771; ABclonal), vimentin (cat. no. ARG66199; Arigo Biolaboratories, Hsinchu, Taiwan), E-cadherin (cat. no. 3195; Cell Signaling), phospho-histone H2AX-Ser139 (cat. no. 2577; Cell Signaling), caspase-3 (cat. no. MBS8811560; MyBioSource, San Diego, CA, USA), GAPDH (cat. no. 2118; Cell Signaling), and ACTB (β-actin; cat. no. 4968; Cell Signaling) overnight at 4 °C. After washing 3 times with Tris-buffered saline with 1% Tween-20, the membranes were hybridized with horseradish peroxidase-conjugated secondary antibodies at room temperature for 1 h. Protein expression was visualized by an enhanced chemiluminescence substrate (Millipore, Billerica, MA, USA) and imaged using a BioSpectrum Imaging System (UVP, Upland, CA, USA). The intensities of bands were analyzed using ImageJ 1.48v (National Institutes of Health, USA).

### RNase R treatment

To confirm the circular structure of *circAAGAB*, total RNA was treated with 3 U RNase R (Lucigen, LGC Ltd, Teddington, UK) and 10X reaction buffer (Lucigen), and then incubated at 37 °C for 20 min. After RNA reverse transcription and PCR, PCR products were subjected to gel electrophoresis and visualized by a UVP Gel Solo system (Analytik Jena US, Upland, CA, USA).

### RNA pull-down assay

A total of 1 × 10^7^ MDA-MB-231 cells were lysed by cell lysis buffer (25 mM Tris–HCL pH 7.5, 150 mM NaCl, 1 mM EDTA, 0.5% NP-40) for each sample. Then, a Pierce Magnetic RNA–Protein Pull-Down Kit (Thermo Fisher) was used according to the manufacturer’s protocols. Magnetic beads conjugated with streptavidin were incubated with 3 μg biotinylated DNA oligo probe for 1 h. After the complex was formed, cell lysate was added into tubes containing the beads and incubated for 4 h. Subsequently, the complex was washed 4 times with wash buffer from the kit and 500 μL of cell lysis buffer. Finally, washed samples were measured by western blotting.

### Actinomycin D treatment

At approximately 60% confluency, MDA-MB-231 cells were transferred into 6-well plates. Cells were treated with 5 μg/mL actinomycin D (Sigma, Saint Louis, MO, USA) dissolved in DMSO and collected at the indicated time points. Total RNA was purified after the cells were lysed. After treatment with actinomycin D, the RNA expression levels of *circAAGAB* were analyzed by quantitative RT-PCR.

### Nuclear-cytoplasmic fractionation

For nuclear-cytoplasmic fractionation, RNAs in 8 × 10^5^ cells were extracted by a Cytoplasmic & Nuclear RNA Purification Kit (Norgen Biotek Corp., Ontario, Canada) according to the manufacturer’s protocols. The isolated RNA was detected by quantitative RT-PCR and normalized to *GAPDH* (cytoplasmic control) or *BCAR4* (nuclear control).

### RNA fluorescence in situ hybridization (RNA-FISH)

MDA-MB-231 cells (6 × 10^5^) were first seeded in cover glass chamber (80826, ibidi, Martinsried, Germany) overnight, washed with PBS once, and fixed with 4% paraformaldehyde for 10 min. After washing with PBS twice, cells were dehydrated with 70% EtOH for 2 h, and incubated with RNA probe (125 nM) at 37 °C overnight. The RNA labeling probes conjugated with 5ʹ modification 6-FAM (TTC CAA GGA TAT CAT TCT TCA TCA) were designed to target the back-splicing site of *circ-AAGAB*. Next, the cells were washed at 37 °C for 30 min, and mounted with Mounting Medium containing DAPI (ab104139, Abcam, Cambridge, UK). Finally, the images were acquired using a ZEISS LSM880 laser confocal microscopy (ZEISS, Heidelberg, Germany).

### Luciferase reporter assay

HEK293T cells were cultured in 24-well plates with 4 × 10^4^ cells per well. Cells were co-transfected with 50 μg of pMIR-REPORT-*circAAGAB* or pMIR-REPORT-*circAAGAB*-mut, 2 × 10^–2^ ng of *miR-378 h* mimics or mimic control, and 1 μg of Renilla luciferase vector as the transfection control. After transfecting for 48 h, cells were lysed by 100 μL luciferase lysis buffer (92.8 mM K_2_HPO_4_, 9.2 mM KH_2_PO_4_ and 0.2% Triton X-100 in ddH_2_O) on ice for 15 min and then centrifuged at 12,000xg relative centrifugal force for 2 min at 4 °C. Afterwards, the supernatant was isolated, and the luciferase signal was measured using the Dual-Glo Luciferase Assay System (Promega, Fitchburg, WI, USA).

### Microarray analysis

MDA-MB-231 cells were transfected with 4 μg of pCIRC2T7-*circAAGAB* plasmids and total RNA was purified. Subsequently, microarray experiments were done through the service of the Core Instrument Center, National Health Research Institutes (Miaoli, Taiwan). Briefly, human single-stranded cDNA was generated from the amplified complementary RNA with the WT Plus cDNA Synthesis Kit (Affymetrix, Santa Clara, CA, USA), and then fragmented and labeled with the WT Terminal Labeling Kit (Affymetrix). RNA expression profiling was performed using the Clariom S Assay (Affymetrix). After scanning, the data from the Affymetrix microarray was normalized by robust multichip averaging. Visual representation of expression profiles was evaluated by principal component analysis (PCA) and hierarchical clustering by the Genesis 1.7.7 program (Graz University of Technology, Graz, Austria). Interactions between genes, biological functions, and pathways were analyzed by Ingenuity Pathway Analysis (IPA, Ingenuity Systems Inc., Redwood City, USA). The datasets generated during the current study are available in the Gene Expression Omnibus repository (https://www.ncbi.nlm.nih.gov/geo/query/acc.cgi?acc=GSE158779).

### Colony formation assay

MDA-MB-231 cells were cultured in 6-well plates with 2 × 10^5^ cells per well and transfected with 4 μg of pCIRC2T7-*circAAGAB* plasmids for 24 h. Cells were reseeded into 6-well plates with 500 cell per well. After 3 weeks, cells were fixed by fixing buffer containing 75% methanol and 25% acetate (Sigma), and 0.1% crystal violet (Sigma) was added to dye the cells. Colonies of at least 50 cells were counted manually.

### Cell migration and invasion assay

MDA-MB-231 cells were seeded in 6-well plates with 2 × 10^5^ cells per well and transfected with pCIRC2T7-*circAAGAB* plasmids for 24 h. For cell migration, 6 × 10^5^ cells were seeded into the transwell chambers and incubated for 24 h. For cell invasion, 1 × 10^6^ cells were seeded into the transwell chambers coated with Matrigel and incubated for 48 h. After migration and invasion, cells were fixed by fixing buffer containing 75% methanol and 25% acetate (Sigma), and 0.1% crystal violet (Sigma) was added to stain the cells. Cells on the membrane were counted manually.

### Ionizing radiation (IR) treatment

MDA-MB-231 cells were seeded and transfected with pCIRC2T7-*circAAGAB* plasmids for 24 h. Then, the cells were exposed to 10 Gy of γ-rays by an IBL-637 Cesium-137 γ-ray machine (Cis-Bio International, Ion Beam Applications, Belgium) and harvested at 24 h after irradiation. Finally, the cells were stained by propidium iodide (PI) and analyzed on a Beckman Coulter FC500 instrument (Beckman Coulter, Inc.) using CXP Analysis Software v2.3 (Beckman Coulter, Inc.).

### Cell apoptosis and cell cycle analysis

MDA-MB-231 cells were seeded in 10 cm dishes with 1 × 10^6^ cells per dish and transfected with pCIRC2T7-*circAAGAB* plasmids for 24 h. Cells for examining apoptosis were harvested and stained by using a FITC Annexin V Apoptosis Detection Kit I (PharMingen, BD Biosciences, NJ, USA) according to the manufacturer’s protocols. Cells for examining the cell cycle were harvested and fixed by 75% ethanol at − 20 °C overnight, then lysed with 0.5% Triton X-100 (Amersham, Little Chalfont, Buckinghamshire, UK), subjected to RNase A (Qiagen) treatment (20 ng RNase A/mL in PBS) and stained with PI (BD Biosciences, NJ, USA) solution (20 μg PI/mL in PBS) in the dark for 10 min. Afterwards, cell cycle and cell apoptosis were detected by a Beckman Coulter FC500 instrument (Beckman Coulter, Inc.) using CXP Analysis Software v2.3 (Beckman Coulter, Inc.).

### Statistical analysis

All quantitative data are presented as the means ± standard deviations of data from at least three independent experiments, and an unpaired Student’s t-test was used to compare differences between groups. All analyses were performed using Microsoft Office Excel software, and a *P*-value < 0.05 was considered to be statistically significant.

## Results

### Characterization of hypoxia-responsive circular RNA *circAAGAB*

**Fig. 1:**
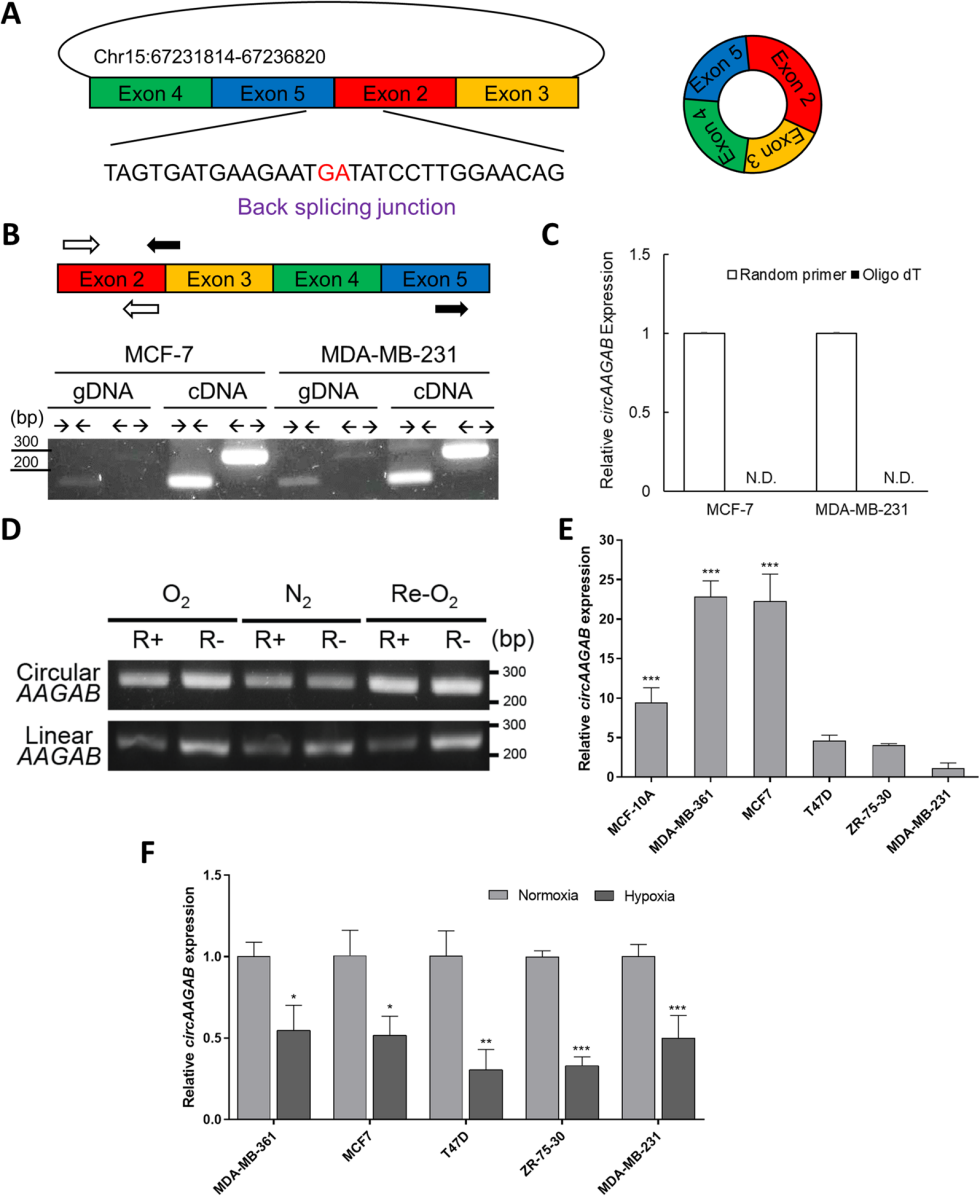
Characterization of hypoxia-responsive circular RNA *circAAGAB* in breast cancer cells under different oxygen concentrations. **A** Sequence of back-splicing junction using CircRNA Identifier (CIRI) [35]. **B** Gel electrophoresis of PCR products. Divergent primers (black arrows; ← →) and convergent primers (white arrows; → ←) for PCR. Genomic DNA (gDNA) or complementary DNA (cDNA) was used as the template to examine endogenous *circAAGAB* in MCF-7 and MDA-MB-231 cells. The PCR product length is 248 bp using divergent primers and 159 bp by using convergent primers. **C** Relative expression levels of *circAAGAB* by quantitative RT-PCR. Random primers or oligo dT were used for RT-PCR. N.D.: not detected. **D** Gel electrophoresis of PCR products from MCF-7 cells treated with RNase R under different oxygen concentrations. MCF-7 cells were treated with 3U RNase R for 20 min under normoxia (O_2_), hypoxia (N_2_), or re-oxygenation (Re-O_2_). The PCR product length of circular *AAGAB* is 248 bp and length of linear *AAGAB* is 254 bp. **E** Endogenous expression levels of *circAAGAB* in 5 breast cancer cell lines and normal breast epithelial MCF-10A cells by quantitative RT-PCR. **F** Quantitative RT-PCR analysis of *circAAGAB* in 5 breast cancer cell lines under normoxia and hypoxia. All of the quantitative RT-PCR results were normalized to the internal control 18S. *P < 0.05, **P < 0.01, ***P < 0.001

Since hypoxia promotes the malignancy of solid tumors, in order to determine whether circRNA acts as a tumor suppressor in breast cancer, hypoxia-downregulated circRNAs were explored by RNA sequencing from MCF-7 cells growing under normoxia (O_2_), hypoxia (N_2_), and re-oxygenation (Re-O_2_) conditions. The transcriptome of different oxygen conditions was profiled by Illumina sequencing after depleting ribosomal RNA [34]. CircRNA Identifier (CIRI) [35] was used to predict putative circRNAs by identifying the sequence of the back-splicing junction. The criteria for selecting hypoxia-downregulated circRNAs consisted of significant differences (*P*-value < 0.05) between N_2_ and O_2_ conditions and N_2_ and Re-O_2_ conditions, as well as no significant differences (*P*-value > 0.05) between O_2_ and Re-O_2_ conditions. *CircAAGAB*, consisting of exons 2 to 5 of *AAGAB* (5,007 nucleotides) (Fig. 1), was chosen for further experiments because it was down-regulated the most under hypoxia as compared with normoxia.

Firstly, to validate this circRNA by PCR, divergent primers (black arrows) were designed for priming at the back-splicing junction of *circAAGAB*; convergent primers (white arrows) were designed at exon 2 for both circular and linear *AAGAB* (Fig. 1B). gDNA and cDNA were used as the template in MCF-7 and MDA-MB-231 cells. The results showed the existence of linear *AAGAB* in gDNA and cDNA by using convergent primers (**→ ←**), and that circular *AAGAB* could only be amplified by using divergent primers (← →) in cDNA, not gDNA (Fig. 1B). In addition, given that the absence of a polyA tail is one of the characteristics of circRNAs, random primers and oligo dT primers were used to reverse-transcribe total RNA to cDNA, followed by quantitative RT-PCR. The results showed that only random primers, not the oligo dT primer, were able to amplify *circAAGAB* (Fig. 1C). Fourthly, total RNA was extracted from MCF-7 cells under O_2_, N_2_, and Re-O_2_ conditions, and was treated with 3 U of RNase R, an enzyme to digest linear RNAs. The product amplified by the divergent primer (*circAAGAB*) maintained the same expression level through RNase R treatment, but linear *AAGAB* was degraded in the presence of RNase R (Fig. 1D).

To determine the endogenous expression of *circAAGAB* in breast cancer cells, MDA-MB-361, MCF-7, T47D, ZR-75-30 and MDA-MB-231 cells, and human breast epithelial cell line MCF-10A cells were examined by quantitative RT-PCR. The results revealed that MDA-MB-231 cells had the least endogenous expression of *circAAGAB* (Fig. 1E). Furthermore, *circAAGAB* was significantly (*P* < 0.05) down-regulated under hypoxia in all examined breast cancer cell lines (Fig. 1F). These data suggested that the endogenous *circAAGAB* was downregulated under hypoxia in breast cancer cells.

### Identification of RNA binding protein of circAAGAB

**Fig. 2:**
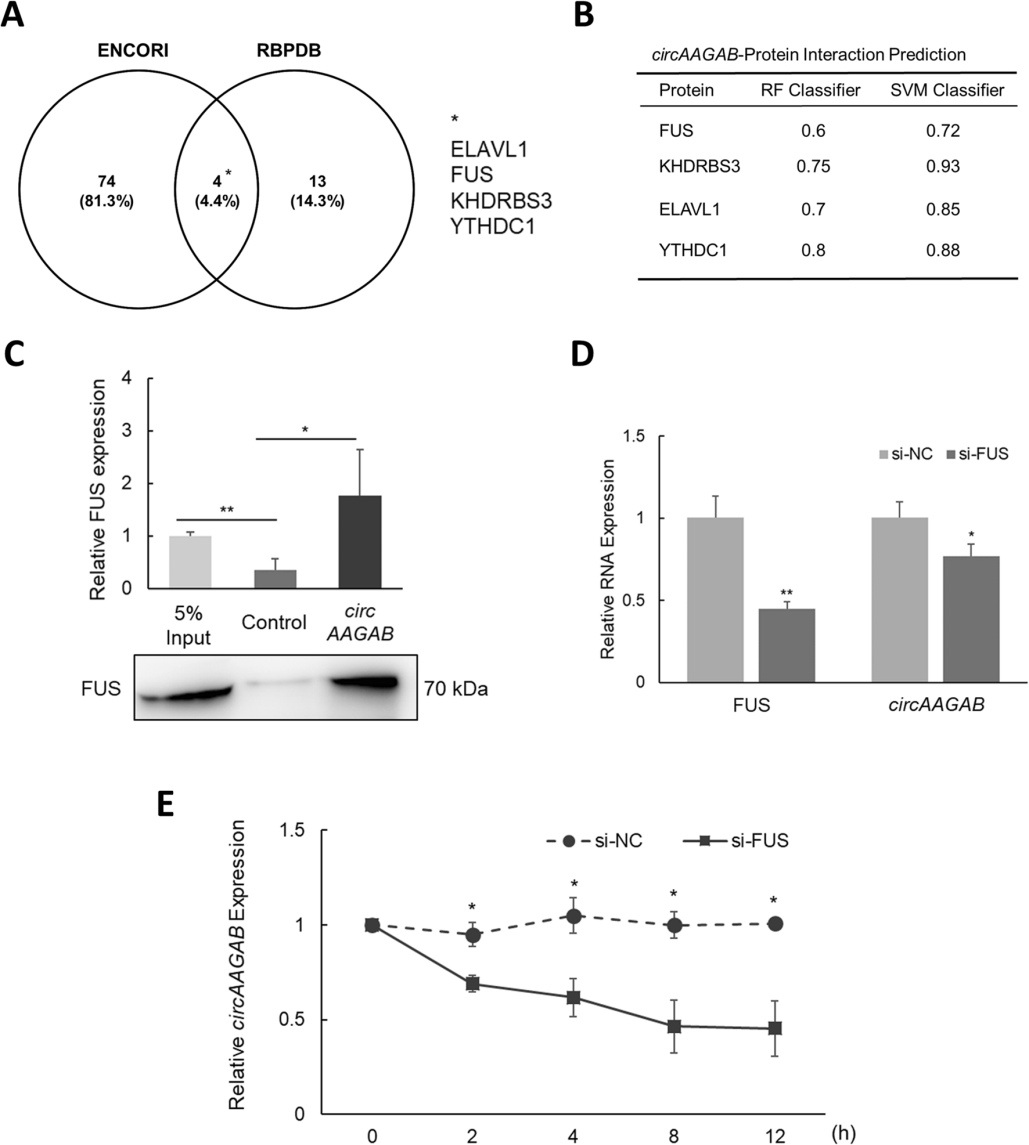
RNA binding protein FUS increases the stability of *circAAGAB* via direct binding. **A** Venn diagram of the predicted RNA binding proteins (RBPs). RNA binding proteins with *circAAGAB* were predicted by ENCORI (http://starbase.sysu.edu.cn/) and RBPDB (http://rbpdb.ccbr.utoronto.ca/). * The 4 common RBPs are shown. **B** The predicted possibility of interaction between *circAAGAB* and RBP. The binding possibility was calculated by RNA–Protein Interaction Prediction (RPISeq) (http://pridb.gdcb.iastate.edu/RPISeq/) using random forest (RF) and support vector machine (SVM) classifiers. Predictions with probabilities > 0.5 were considered positive. **C** RNA pull-down assay. Cell lysates were extracted from MDA-MB-231 cells, pulled down by biotin-labeled probe against *circAAGAB*, and subjected to western blotting using FUS antibody. **D** Expression levels of RNA in MDA-MB-231 cells transfected with siRNA against *FUS* by quantitative RT-PCR. NC: nontargeting control siRNA. Internal control: *GAPDH*. **E** Stability assay of *circAAGAB*. MDA-MB-231 cells were transfected with 5 μg/mL actinomycin D and siRNA against *FUS.* The expression levels of *circAAGAB* were measured by quantitative RT-PCR at various time points. NC: nontargeting control siRNA. Internal control: *GAPDH*. *P < 0.05, **P < 0.01

Next, to identify RBPs interacting with *circAAGAB*, the bioinformatics tools, Encyclopedia of RNA Interactomes (ENCORI; (http://starbase.sysu.edu.cn/) and RNA-Binding Protein DataBase (RBPDB; http://rbpdb.ccbr.utoronto.ca/) were used to predict putative RBPs. ELAVL1, FUS, KHDRBS3, and YTHDC1 were the commonly predicted RBPs (Fig. 2A). Also, RNA-Protein Interaction Prediction (RPISeq) (http://pridb.gdcb.iastate.edu/RPISeq/) predicted the possible binding between *circAAGAB* and the four RBPs (Fig. 2B). In this study, FUS was chosen to explore whether it binds with *circAAGAB*. Firstly, RNA pull-down assays using a biotin-labeled probe against the junction of exons 2 and 5 of *circAAGAB* were performed in MDA-MB-231 cells. The results of western blotting illustrated that FUS could be pulled down by the probe specifically targeting circular *AAGAB*, but not by control probe (Fig. 2C). Next, to explore the effects of FUS on *circAAGAB*, *FUS* was knocked down by siRNA in MDA-MB-231 cells, and this significantly (*P* <0.05) decreased the expression of *circAAGAB* (Fig. 2D). Lastly, to explore the mechanism by which *FUS* silencing down-regulated *circAAGAB* in MDA-MB-231 cells, the stability of *circAAGAB* was examined. MDA-MB-231 cells were transfected with siRNA against *FUS* and simultaneously treated with actinomycin D, a transcription inhibitor, and then the expression levels of *circAAGAB* were examined at 0, 2, 4, 8, 12 h, respectively. The results displayed that the expression levels of *circAAGAB* were decreased significantly (*P* <0.05) after silencing *FUS* (Fig. 2E). These findings suggested that FUS increased the stability of *circAAGAB* through direct binding.

### Identification of microRNA sponged with *circAAGAB*

**Fig. 3:**
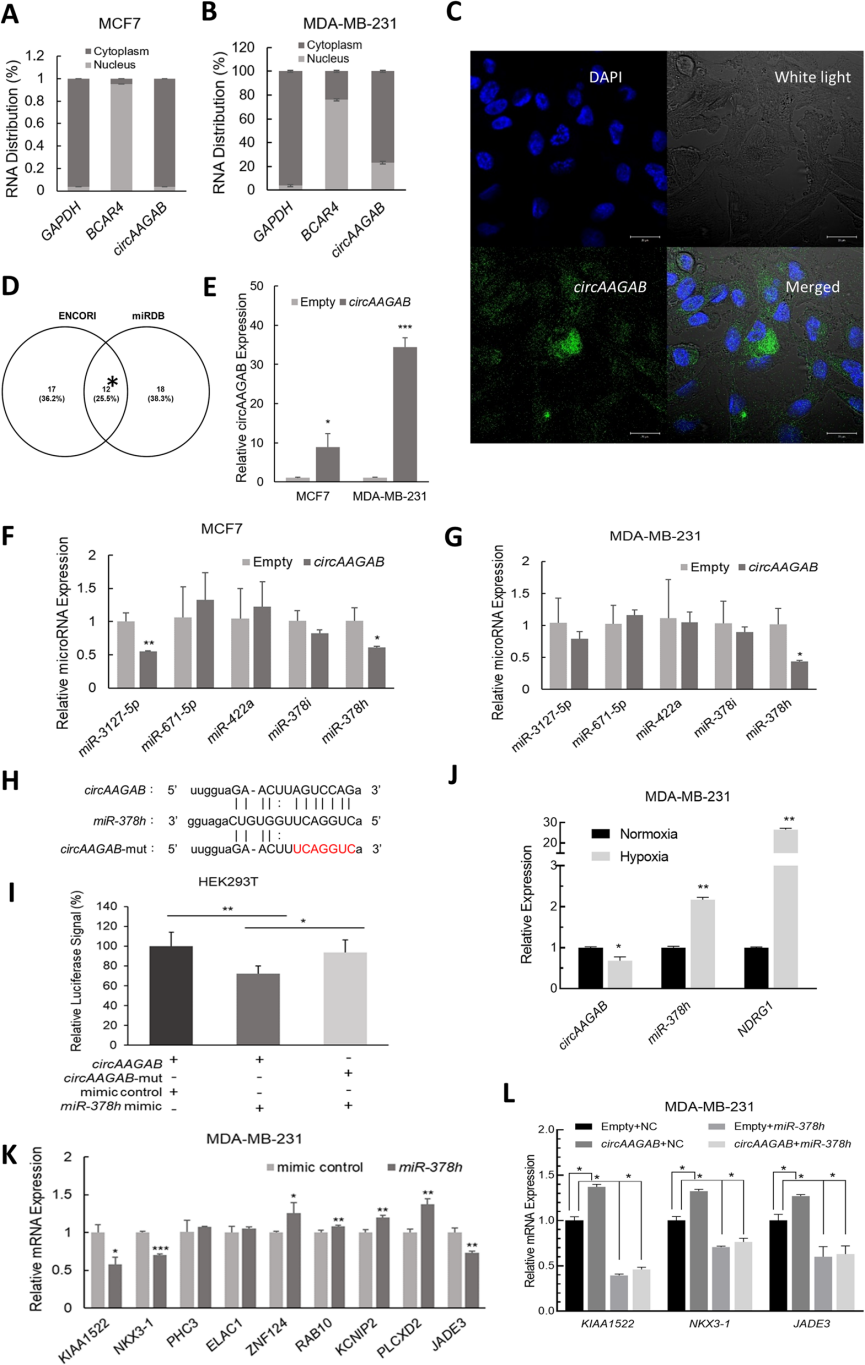
*CircAAGAB* acts as a sponge for *miR-378 h* and inhibits its effect on target genes. **A** Nucleus-cytoplasm fractionation of RNA in MCF-7 cells under hypoxia. Distribution of *circAAGAB* in MCF-7 cells was detected by quantitative RT-PCR. *GAPDH*: cytoplasmic marker; *BCAR4*: nuclear marker. **B** Nucleus-cytoplasm fractionation of RNA in MDA-MB-231 cells under hypoxia. C RNA fluorescence in situ hybridization. Probe labelled with digoxigenin-conjugated UTP was designed to hybridize *circAAGAB*. DAPI: nucleus marker. Scale bar: 20 μm. **D** Venn diagram of the predicted miRNAs. The *circAAGAB*-binding miRNA candidates were predicted by ENCORI (http://starbase.sysu.edu.cn/) and miRDB (http://mirdb.org/). * Top 5 miRNAs with the highest target score of the 12 common miRNAs are shown. **E** Expression levels of *circAAGAB* in cells overexpressing *circAAGAB* by quantitative RT-PCR. MCF-7 and MDA-MB-231 cells were transfected with 4 μg *circAAGAB* plasmids (pCIRC2T7-*circAAGAB*). Internal control: 18S rRNA. **F**, **G** Expression levels of miRNAs in MCF-7 (**F**) or MDA-MB-231 (**G**) cells overexpressing *circAAGAB*. The top 5 miRNAs with highest target score from miRDB were chosen for validation by quantitative RT-PCR. Internal control: 18S rRNA. **H** Schematic graph of the potential binding site of *circAAGAB* on *miR-378 h*. The binding site was aligned by ENCORI. Letters in red are the mutation site. **I** Luciferase reporter assay. HEK293T cells were co-transfected with pMIR-REPORT-*circAAGAB* or its mutant plasmid, *miR-378 h* mimics, and Renilla plasmid for 48 h. The luciferase signal was measured by the Dual-Glo luciferase reporter assay system. The firefly signal was normalized to Renilla signal and mimic control. **J** Expression levels of *circAAGAB* and *miR-378 h* in MDA-MB-231 cells under hypoxia. *NDRG1*: positive control of hypoxia. **K** Expression levels of the predicted target genes of *miR-378 h* in cells overexpressing *miR-378 h*. MDA-MB-231 cells were transfected with *miR-378 h* mimics. RNA levels of the target genes predicted by miRDB and DIANA (http://diana.imis.athena-innovation.gr/DianaTools/index.php) were measured by quantitative RT-PCR. Internal control: *GAPDH*. **L** Expression levels of the downstream genes in MDA-MB-231 cells overexpressing *circAAGAB* and/or *miR-378 h* by quantitative RT-PCR. *P < 0.05, **P < 0.01, ***P < 0.001

Next, to explore the regulatory roles of *circAAGAB* in breast cancer cells, nucleus-cytoplasm fractionation for RNA was performed in MCF-7 and MDA-MB-231 cells under hypoxia. The results displayed that *circAAGAB* was mostly distributed in the cytoplasm under hypoxia in both MCF-7 (Fig. 3A) and MDA-MB-231 cells (Fig. 3B). RNA-FISH assay also indicated that the location of *circ-AAGAB* was mainly at cytoplasm (Fig. 3C). Since circRNAs in cytoplasm were reported to serve as miRNA sponges, the potential miRNA targets were predicted by ENCORI and miRDB (http://mirdb.org/). Among the predicted miRNAs, there were 12 miRNAs in common (Fig. 3D). The top 5 miRNAs with the highest target score from miRDB analysis (*miR-3127-5p*, *miR-671-5p*, *miR-422a*, *miR-378i*, and *miR-378 h*) were chosen for experimental validation (Fig. 3D). First, MCF-7 and MDA-MB-231 cells were transfected with the vector, pCIRC2T7-*circAAGAB*, to overexpress *circAAGAB*. After *circAAGAB* was successfully overexpressed in MCF-7 and MDA-MB-231 cells (Fig. 3E), the expression levels of the predicted miRNAs were measured in MCF-7 (Fig. 3F) and MDA-MB-231 (Fig. 3G) cells overexpressing *circAAGAB.* Among the sponged miRNAs, the results showed that only *miR-378 h* expression was significantly (*P* < 0.05) reduced in both MCF-7 (Fig. 3F) and MDA-MB-231 (Fig. 3G) cells overexpressing *circAAGAB*. To further verify the direct binding of *circAAGAB* to *miR-378 h*, the luciferase reporter plasmid, pMIR-REPORT-*circAAGAB*, was constructed, and the potential binding site of *miR-378 h* on *circAAGAB*, i.e., the seed region of *miR-378 h*, was mutated (Fig. 3H). When HEK293T cells were co-transfected with pMIR-REPORT-*circAAGAB* and *miR-378 h* mimics, the luciferase signals were significantly (*P* < 0.01) reduced, and this result was reversed in the presence of the *circAAGAB* mutant (Fig. 3I). Furthermore, as the expression of *circAAGAB* was downregulated under hypoxia, the expression levels of *miR-378 h* were upregulated in MDA-MB-231 cells under hypoxia (Fig. 3J).

Next, to investigate the target genes of *miR-378 h*, miRDB and DIANA (http://diana.imis.athena-innovation.gr/DianaTools/index.php) were first used to predict the target genes. The top 9 genes with the highest target scores from the miRDB analysis were examined in MDA-MB-231 cells overexpressing *miR-378 h*. The RNA levels of only three genes, *KIAA1522*, *NKX3-1,* and *JADE3*, were significantly (*P* < 0.05) decreased (Fig. 3K). Subsequently, quantitative RT-PCR was performed to confirm whether *KIAA1522*, *NKX3-1,* and *JADE3* were downstream genes of *circAAGAB*. As the results show, *KIAA1522*, *NKX3-1,* and *JADE3* expression significantly (*P* < 0.05) increased in MDA-MB-231 cells overexpressing *circAAGAB*, and downregulated in cells overexpressing *miR-378 h* alone or together with *circAAGAB* (Fig. 3L). These results suggested that *miR-378 h* was the downstream gene of *circAAGAB* and *circAAGAB* disinhibited the target genes of *miR-378 h*, *KIAA1522*, *NKX3-1,* and *JADE3*, by sponging *miR-378 h* (Fig. 3L).

### Identification of *circAAGAB*-regulated genes

**Fig. 4:**
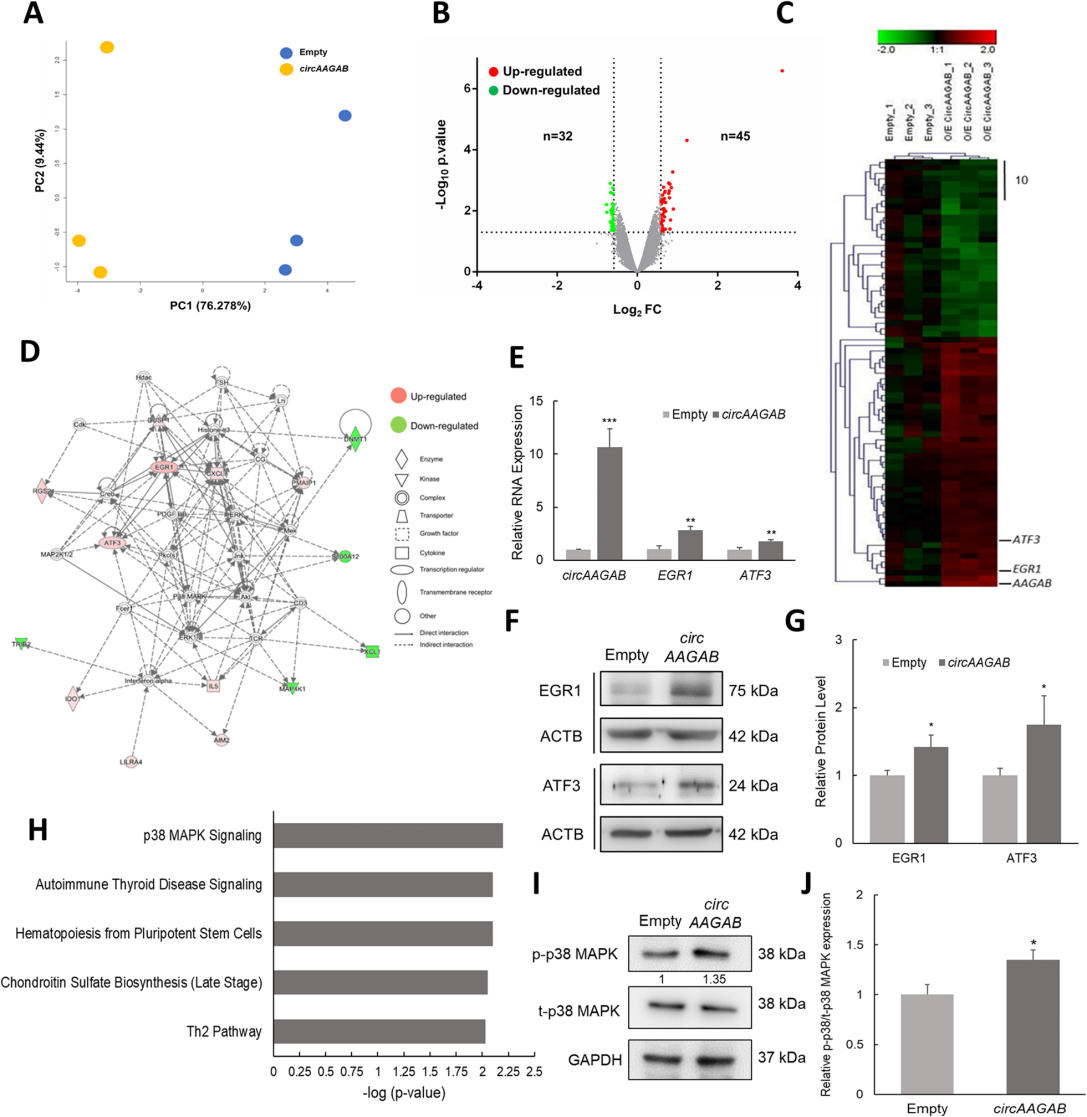
C*ircAAGAB*-regulated genes are involved in the p38 MAPK signaling pathway. **A** Principal component analysis (PCA) of samples overexpressing *circAAGAB*. PCA was plotted by the expression profiles of differentially expressed probes after quantile normalization. Yellow dots represent the *circAAGAB*-overexpressing group, and blue dots represent the empty vector group. **B** Volcano plot of differentially expressed genes in MDA-MB-231 cells overexpressing *circAAGAB* by Affymetrix microarray. Horizontal dashed line corresponds to *P* = 0.05. Vertical dashed lines correspond to fold change (FC) > 1.5X. **C** Heat map of differentially expressed genes. Red: higher expression levels as compared to the average of this gene in the empty vector group. Green: lower expression levels. Scale bar: 10 genes. **D** A representative network of *circAAGAB* downstream genes by Ingenuity Pathway Analysis. **E** Expression levels of *EGR1* and *ATF3* in MDA-MB-231 cells overexpressing *circAAGAB* by quantitative RT-PCR. Internal control: *GAPDH*. **F** Western blotting of EGR1 and ATF3 protein in MDA-MB-231 cells overexpressing *circAAGAB*. Loading control: ACTB. **G** Quantification of EGR1 and ATF3 protein levels in MDA-MB-231 cells overexpressing *circAAGAB*. **H** Canonical pathways analysis of *circAAGAB* downstream genes. **I** Western blotting for the phosphorylated (p) p38 MAPK in MDA-MB-231 cells overexpressing *circAAGAB*. t: total protein. **J** Quantification of phosphorylated (p) p38 MAPK in MDA-MB-231 cells overexpressing *circAAGAB*. Phosphorylated p38 MAPK was normalized to total (t) p38 MAPK. *P < 0.05, **P < 0.01, ***P < 0.001

To investigate the cellular function of *circAAGAB* in breast cancer cells, differentially expressed genes were first identified in MDA-MB-231 cells overexpressing *circAAGAB* by Affymetrix microarrays. The genomic profiling of MDA-MB-231 cells overexpressing *circAAGAB* was evaluated by PCA. As shown in Fig. 4A, the distribution between *circAAGAB*-overexpressing samples (yellow dots) and the empty control samples (blue dots) was separated, indicating the different transcription profiling in MDA-MB-231 cells overexpressing *circAAGAB*. The criteria we used to define differentially expressed genes were a ≧1.5-fold change and a *P*-value < 0.05. In total, 77 differentially expressed genes were identified, 45 up-regulated and 32 down-regulated genes (Fig. 4B, C). Next, IPA was used to analyze the functions which the *circAAGAB*-regulated genes were involved in. One of the representative networks showed that the *circAAGAB*-regulated genes were involved in cell death and survival (Fig. 4D). In this network, 2 hub genes, *EGR1* and *ATF3*, which had the highest fold changes in the microarray data, were validated by quantitative RT-PCR (Fig. 4E) and western blotting (Fig. 4F&G). Both showed significant (*P* < 0.01) up-regulation in MDA-MB-231 cells overexpressing *circAAGAB* (Fig. 4E–G). In addition, results of canonical pathway analysis revealed that *circAAGAB* downstream genes participated in the p38 MAPK signaling pathway (Fig. 4H). The results of western blotting indeed showed that phosphorylated p38 MAPK was significantly (*P* < 0.05) activated in MDA-MB-231 cells overexpressing *circAAGAB* (Fig. 4I, J). These results indicated that *circAAGAB* regulated apoptosis-related genes, *EGR1* and *ATF3*, and the p38 MAPK signaling pathway.

### Identification of function of *circAAGAB*

**Fig. 5:**
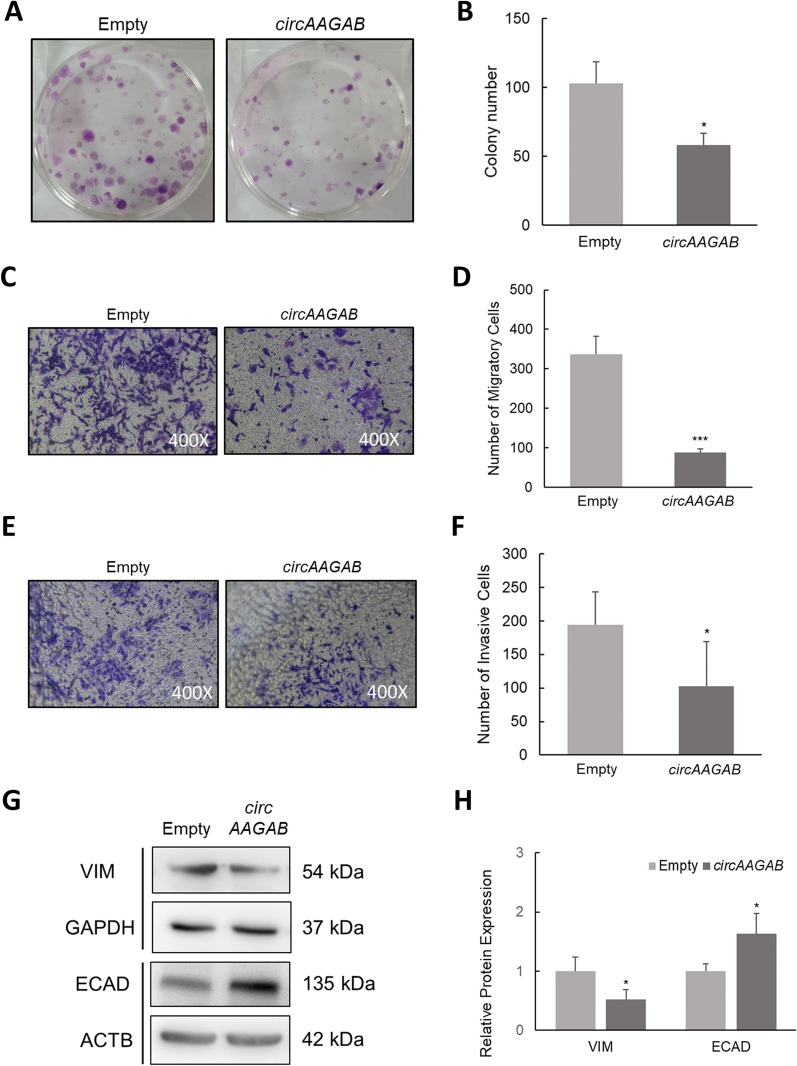
*CircAAGAB* inhibits colony formation, cell migration, invasion and epithelial-mesenchymal transition in MDA-MB-231 cells. **A** Colony formation assay in MDA-MB-231 cells overexpressing *circAAGAB*. MDA-MB-231 cells were transfected with *circAAGAB* plasmids, seeded into 6-well dishes, and incubated for 3 weeks. **B** Quantification of colony formation assay. **C** Migration assays of MDA-MB-231 cells overexpressing *circAAGAB*. Cells were transfected with *circAAGAB* plasmids, and 6 × 10^5^ cells were seeded into the inserts. The image was taken at 24 h after seeding. **D** Quantification of the migrating cells by transwell assays. **E** Invasion assays of MDA-MB-231 cells overexpressing *circAAGAB*. Cells were transfected with *circAAGAB* plasmids, and 1 × 10^6^ cells were seeded into the inserts with Matrigel coating on the membrane. The image was taken at 48 h after seeding. **F** Quantification of the invasive cells by transwell assays. **G** Western blotting for epithelial–mesenchymal transition markers vimentin (VIM) and E-cadherin (ECAD) in MDA-MB-231 cells overexpressing *circAAGAB*. Loading control for VIM: GAPDH. Loading control for E-cadherin ECAD: ACTB. **H** Quantification of VIM and ECAD in MDA-MB-231 cells overexpressing *circAAGAB*. The expression values were normalized to the respective loading controls. *P < 0.05, ***P < 0.001

Lastly, in vitro functional assays were performed. To determine the effects of *circAAGAB* on cell proliferation and colony formation, BrdU and MTT assays (data not shown) were conducted in MDA-MB-231 cells. The results indicated that *circAAGAB* inhibited colony formation (Fig. 5A, B), but had no effect on short-term cell proliferation by BrdU assays and MTT assays (data not shown). In addition, transwell assays were performed to evaluate cell migration (Fig. 5C, D) and invasion (Fig. 5E, F). The data illustrated that migratory and invasive cells were significantly (*P* < 0.05) decreased in MDA-MB-231 cells overexpressing *circAAGAB* (Fig. 5C–F). Also, the EMT markers vimentin (VIM) and E-cadherin (ECAD), were further measured. As expected, vimentin (mesenchymal marker) expression was significantly (*P* < 0.05) down-regulated, and E-cadherin (epithelial marker) was significantly (*P* < 0.05) up-regulated after overexpressing *circAAGAB* (Fig. 5G, H). These results suggested that *circAAGAB* played the role of tumor suppressor, inhibiting colony formation, cell migration, invasion, and EMT in MDA-MB-231 cells.

**Fig. 6:**
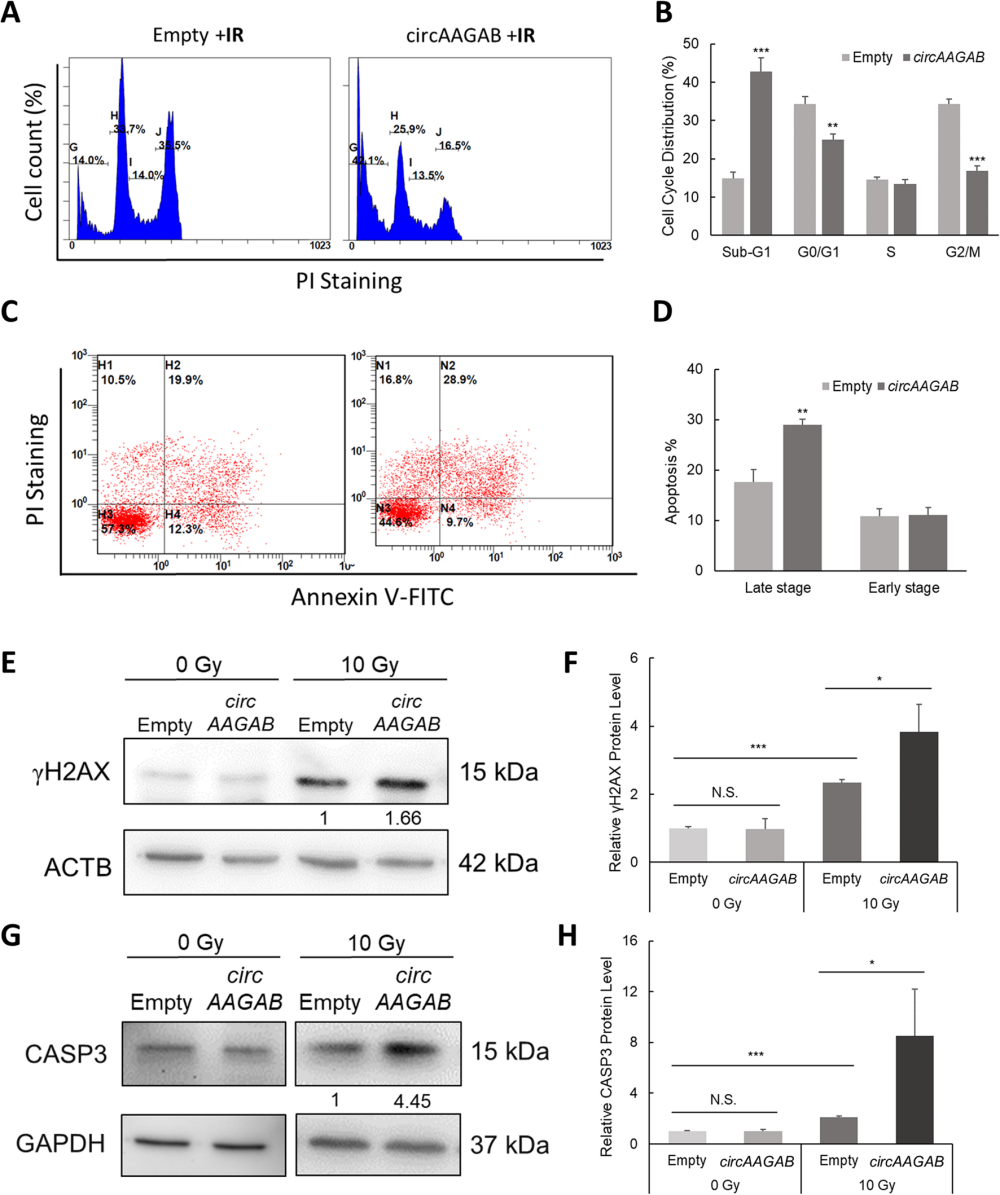
*CircAAGAB* increases radiosensitivity in MDA-MB-231 cells. **A** Representative diagram of flow cytometry in MDA-MB-231 cells overexpressing *circAAGAB* and treated with ionizing radiation (IR). Cells were transfected with *circAAGAB* plasmids and exposed to 10 Gy IR. Cells were then collected at 24 h after IR, fixed with 75% ethanol overnight, and stained with PI. **B** Quantification of cell cycle and apoptotic cells (sub-G1). **C** Representative diagram of flow cytometry with annexin-V and PI staining. Cells were transfected with *circAAGAB* plasmids and exposed to 10 Gy IR. Cells were then collected at 24 h after IR. **D** Quantification of early and late stages of apoptosis in MDA-MB-231 cells. **E**, **G** Western blotting of γH2AX (**E**) and caspase-3 (CASP3) (**G**) in MDA-MB-231 cells overexpressing *circAAGAB* and treated with IR. Cells were transfected with *circAAGAB* plasmids and exposed to 10 Gy IR. Cells were collected at 4 h after IR for γH2AX, and 6 h for CASP3. Loading controls: ACTB or GAPDH. **F**, **H** Quantification of γH2AX (**F**) and CASP3 (**H**) in MDA-MB-231 cells overexpressing *circAAGAB*. *P < 0.05, **P < 0.01, ***P < 0.001

**Fig. 7:**
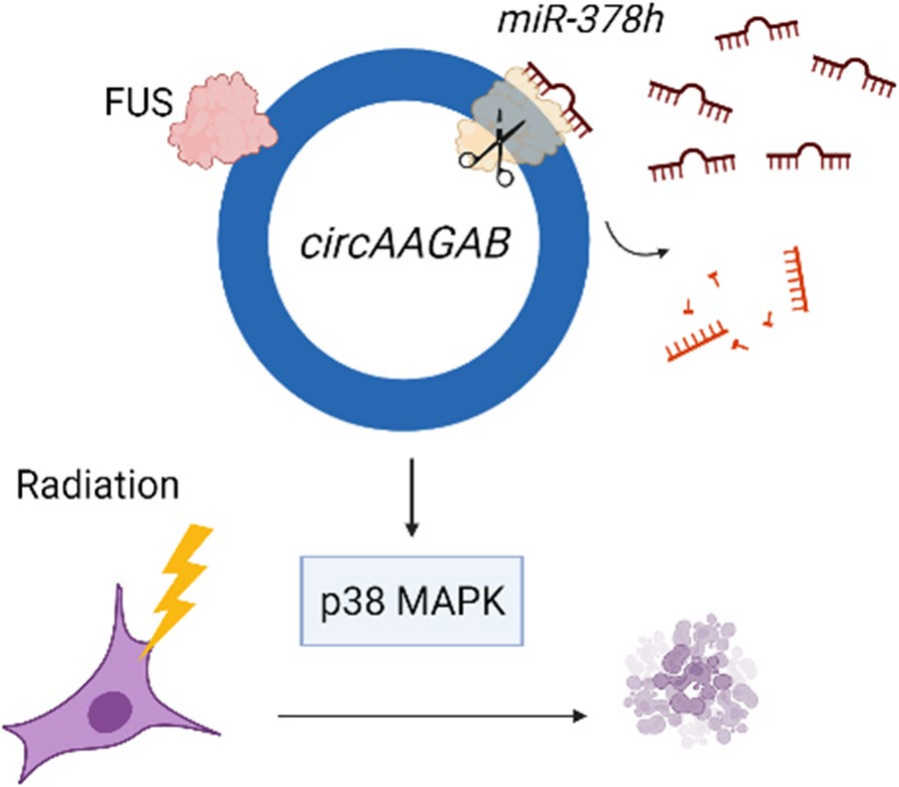
Schematic diagram elucidates the regulatory mechanisms and function of hypoxia-responsive *circAAGAB* in breast cancer cells

As shown in the previous results, *circAAGAB* downstream genes were involved in apoptosis. To determine whether *circAAGAB* regulated cell apoptosis in breast cancer cells, flow cytometry and western blotting were applied in MDA-MB-231 cells overexpressing *circAAGAB*. However, no sign of apoptosis was observed (data not shown). Therefore, we further examined whether *circAAGAB* affected radiosensitivity. As shown in Fig. 6, overexpression of *circAAGAB* in MDA-MB-231 cells after IR significantly (*P* < 0.01) increased the sub-G1 proportion of the cell population (Fig. 6A&B). Similarly, PI staining and annexin V staining illustrated that the percentage of cells in late apoptosis was significantly (*P* < 0.05) increased in MDA-MB-231 cells 24 h after IR (Fig. 6C, D). Subsequently, the marker of DNA damage and repair, γH2AX (Fig. 6E, F), and the apoptosis marker, caspase-3 (Fig. 6G, H), were examined in MDA-MB-231 cells overexpressing *circAAGAB* after IR treatment. The increased amounts of γH2AX (Fig. 6E, F) and caspase-3 (Fig. 6G, H) indicated an inability to repair DNA, resulting in more cell death after IR treatment. These results indicated that *circAAGAB* promoted radiosensitivity in breast cancer cells. All the above results indicated that the hypoxia-responsive circRNA, *circAAGAB*, interacted with FUS, sponged *miR-378 h*, restrained cell colony formation, cell migration and invasion, and increased radiosensitivity in breast cancer cells through the p38 MAPK signaling pathway (Fig. 7).

## Discussion

In this study, the functions and regulatory mechanisms of a novel hypoxia down-regulated circRNA, *circAAGAB*, were identified in breast cancer. First, the circular structure of *circAAGAB* and its expression levels under different oxygen concentrations were validated. Next, we established that the stability of *circAAGAB* increased by binding with the RNA binding protein FUS. Also, *circAAGAB* acted as a sponge of *miR-378 h,* resulting in up-regulation of *KIAA1522*, *NKX3-1,* and *JADE3*, target genes of *miR-378 h*. Finally, *circAAGAB* reduced colony formation and cell migration and invasion via the p38 MAPK pathway, and also increased radiosensitivity in MDA-MB-231 cells.

CircRNAs were initially reported to interact with RBPs in the cytoplasm and to function as protein sponges, protein decoys, and protein scaffolds [36]. For instance, binding of *circPABPN1* with HuR prevented HuR from binding to *PABPN1*, which led to lower translation of PABPN1 [37]. In this study, to evaluate whether *circAAGAB* bound to RBPs, ENCORI and RBPDB (http://rbpdb.ccbr.utoronto.ca/) were used to predict putative RBPs. ELAVL1, FUS, KHDRBS3, and YTHDC1 were the common predicted RBPs (Fig. 2A), and the binding possibility was calculated by RNA–Protein Interaction Prediction (RPISeq) (http://pridb.gdcb.iastate.edu/RPISeq/) (Fig. 2B). Since that KHDRBS3 had less endogenous levels in MDA-MB-231 cells (data not shown), that ELAVL1 might have less possibility of binding on circular RNAs because ELAVL1 bound to AU-rich element of mRNAs [38], and that YTHDC1, a reader of m6A [39], was not predicted in the previous version of ENCORI and was added recently, FUS was selected as the RBP candidate for validation. We discovered that FUS directly bound *circAAGAB* (Fig. 2D) and that knocking down FUS decreased *circAAGAB* stability (Fig. 2E). Recent studies have reported various mechanisms of regulating circRNA levels. For example, circRNA *CDR1as/ciRS-7* was degraded by endonuclease Ago2-mediated cleavage through *miR-671* recruitment [40]. m^6^A-modified circRNAs were shown to be broken down by endoribonucleolytic cleavage through YTHDF2 (m^6^A reader), HRSP12 (adaptor protein), and RNase P/MRP (endoribonucleases) [41]. On the other hand, recent emerging evidence also suggested that RBPs in the nucleus could regulate circuRNA biogenesis. For instance, QKI modulated circRNA formation via binding to specific motifs on the flanking intron of circRNAs [42]. Some studies have reported that FUS modulates circRNA biogenesis by binding to the introns flanking the back-splicing junction [43, 44]. Therefore, we conclude that FUS both regulates the biogenesis of *circAAGAB* in the nucleus and improves *circAAGAB* stability by blocking the excision site of nucleases in the cytoplasm.

Recent evidence suggested that circRNAs can sponge miRNAs to inhibit their effects on target genes, which typically results in up-regulated expression of target genes [18, 19]. In this study, *miR-378 h* was discovered to be down-regulated in two breast cancer cell lines overexpressing *circAAGAB* (Fig. 3F&G), up-regulated in hypoxic cells (Fig. 3J), and the direct binding site of *miR-378 h* on *circAAGAB* was validated by luciferase reporter assays (Fig. 3H&I). Furthermore, the target genes of *miR-378 h*, *KIAA1522*, *NKX3-1,* and *JADE3*, were validated as competing endogenous RNAs of *circAAGAB*, with down-regulation by overexpressing *miR-378 h* (Fig. 3K) and up-regulation by overexpressing *circAAGAB* (Fig. 3L). These findings suggested that *circAAGAB* could act as a sponge for *miR-378 h* to up-regulate the expression levels of *KIAA1522*, *NKX3-1,* and *JADE3*. Although *KIAA1522* was shown to promote malignancy in hepatocellular carcinoma cells [45], *NKX3-1* played the role of tumor suppressor in prostate cancer [46]*,* and *JADE3* was found to increase stemness in colon cancer [47], this is the first discovery of their tumor suppressor roles in breast cancer. However, whether *miR-378 h* can directly bind to *KIAA1522*, *NKX3-1,* and *JADE3* remains unknown and needs to be verified by luciferase assays. Nevertheless, our findings suggested that *circAAGAB* inhibited breast cancer progression through the *circAAGAB*-*miR-378 h*-*KIAA1522/NKX3-1*/*JADE3* axis and could serve as a novel biomarker or target for therapy.

To determine which pathway and cell functions *circAAGAB* was involved in in breast cancer cells, Affymetrix microarrays were performed in MDA-MB-231 cells overexpressing *circAAGAB*. Differentially expressed genes from the microarrays were analyzed, and potential pathways were predicted by IPA and validated by quantitative RT-PCR and western blotting. The results showed that *circAAGAB* up-regulated genes related to cell death and survival, such as *EGR1* and *ATF3* [48, 49] (Fig. 4E–G), and was implicated in the p38 MAPK signaling pathway (Fig. 4H–J). It has been reported that p38 MAPK is involved in many cellular signaling pathways that modulate tumor malignancy, such as proliferation, migration and invasion, EMT, and apoptosis [50–54], and circRNAs could also affect these cell functions in transformed cells. For example, *circ-ITCH* inhibited cell proliferation, colony formation, migration and invasion, and promoted cell apoptosis in bladder cancer via regulating p21 and PTEN expression [55]. In this study, our data revealed that *circAAGAB* could repress colony formation (Fig. 5A, B), cell migration (Fig. 5C, D), and invasion (Fig. 5E, F) in breast cancer. In accordance with this finding, vimentin (mesenchymal marker) expression was down-regulated and E-cadherin (epithelial marker) was up-regulated (Fig. 5G, H).

However, *circAAGAB* enhanced cell apoptosis only in MDA-MB-231 cells treated with IR (Fig. 6). Up-regulation of γH2AX (Fig. 6E, F) and caspase-3 (Fig. 6G, H) expression after IR treatment proved that double-stranded DNA was indeed broken and caused cell death. The remarkable increase of γH2AX (Fig. 6E, F) and caspase-3 (Fig. 6G, H) expression in MDA-MB-231 cells both overexpressing *circAAGAB* and treated with IR suggested that DNA repair capability was decreased and induced cell apoptosis. These results inferred that *circAAGAB* increases radiosensitivity in breast cancer, which is consistent with other evidence that circRNA may affect radiosensitivity. For example, knocking down *circ_0086720* increased radiosensitivity in lung cancer by modulating the *miR-375*/*SPIN1* axis [56].

IR can generate reactive oxygen species (ROS) and causes double-strand DNA breaks, inducing a series of DNA damage responses [57]. As shown in previous studies, double-stranded DNA damage activates ataxia telangiectasia mutated and its downstream proteins, including γH2AX, CHK2, p53, and p21. Via these proteins, activation of ATM results in ROS-triggered p38 MAPK activity and leads to cell cycle arrest or cell apoptosis [58–60]. As shown previously in this study, *circAAGAB* reduced cell colony formation, migration and invasion through the p38 MAPK signaling pathway without radiation treatment. It is possible that *circAAGAB* could activate apoptosis as part of the DNA damage response via the p38 MAPK signaling pathway. However, the downstream cellular pathways of p38 MAPK were not clear, and further experiments are warranted.

In this study, the regulatory mechanism of *circAAGAB* and its effects on cellular functions in MDA-MB-231 cells were determined. Nevertheless, there were some limitations in this research. For example, since MDA-MB-231 cells had the lowest endogenous expression levels and better overexpression efficiency of *circAAGAB* as compared to other breast cancer cell lines, in vitro cellular function assays were performed in MDA-MB-231 cells. Yet, to make the functional role of *circAAGAB* more convincing, the in vitro cellular function assays could be done in other breast cancer cells with high endogenous expression levels of *circAAGAB*, such as MCF-7 or MDA-MB-361 cells, by transfecting siRNA against *circAAGAB*. Furthermore, xenograft assays of MDA-MB-231 cells over-expressing *circAAGAB* in nude mice and examination of the expression levels of *circAAGAB* in clinical specimens may be other possible routes in the future.

## Conclusions

Taken together, this study revealed that a hypoxia-responsive circRNA, *circAAGAB*, interacted with FUS to avoid degradation, sponged *miR-378 h* to up-regulate *KIAA1522*, *NKX3-1*, and *JADE3*, restrained cell colony formation, cell migration and invasion, and increased radiosensitivity in breast cancer cells through the p38 MAPK signaling pathway.

## Data Availability

The microarray datasets generated during the current study are available in the Gene Expression Omnibus repository (https://www.ncbi.nlm.nih.gov/geo/query/acc.cgi?acc=GSE158779).

## Abbreviations

circRNA: Circular RNA
PD-L1: Programmed death 1 ligand
CTLA-4: Cytotoxic T-lymphocyte-associated protein 4
miRNA: MicroRNA
RBP: RNA binding protein
IRES: Internal ribosome entry site
EMT: Epithelial–mesenchymal transition
FBS: Fetal bovine serum
siRNA: Small interfering RNA
gDNA: Genomic DNA
cDNA: Complementary DNA
Ct: Cycle threshold
SDS-PAGE: Sulfate‑polyacrylamide gel electrophoresis
PVDF: Polyvinylidene difluoride
PCA: Principal component analysis
IPA: Ingenuity pathway analysis
IR: Ionizing radiation
PI: Propidium iodide
CIRI: CircRNA Identifier
ENCORI: Encyclopedia of RNA interactomes
RBPDB: RNA-binding protein database
RPISeq: RNA–protein interaction prediction
VIM: Vimentin
ECAD: E-cadherin
RF: Random forest
SVM: Support vector machine
